# Pulmonary embolism response team (PERT) implementation and its clinical value across countries: a scoping review and meta-analysis

**DOI:** 10.1007/s00392-022-02077-0

**Published:** 2022-08-17

**Authors:** Lukas Hobohm, Ioannis T. Farmakis, Karsten Keller, Barbara Scibior, Anna C. Mavromanoli, Ingo Sagoschen, Thomas Münzel, Ingo Ahrens, Stavros Konstantinides

**Affiliations:** 1 Department of Cardiology, Center of Thrombosis and Hemostasis (CTH), University Medical Center of the Johannes Gutenberg-University, Mainz, Germany; 2 Center for Thrombosis and Hemostasis (CTH), University Medical Center of the Johannes Gutenberg University, Mainz, Germany; 3 Medical Clinic VII, University Hospital Heidelberg, Heidelberg, Germany; 4 Department of Cardiology and Medical Intensive Care, Augustinerinnen Hospital, Academic Teaching Hospital University of Cologne, Cologne, Germany; 5 Department of Cardiology, Democritus University of Thrace, Thrace, Greece

**Keywords:** Pulmonary embolism, Pulmonary embolism response team, Advanced therapies, Catheter directed treatment, Systemic thrombolysis

## Abstract

**Background:**

Over the last years, multidisciplinary pulmonary embolism response teams (PERTs) have emerged to encounter the increasing variety and complexity in the management of acute pulmonary embolism (PE). We aimed to systematically investigate the composition and added clinical value of PERTs.

**Methods:**

We searched PubMed, CENTRAL and Web of Science until January 2022 for articles designed to describe the structure and function of PERTs. We performed a random-effects meta-analysis of controlled studies (PERT vs. pre-PERT era) to investigate the impact of PERTs on clinical outcomes and advanced therapies use.

**Results:**

We included 22 original studies and four surveys. Overall, 31.5% of patients with PE were evaluated by PERT referred mostly by emergency departments (59.4%). In 11 single-arm studies (1532 intermediate-risk and high-risk patients evaluated by PERT) mortality rate was 10%, bleeding rate 9% and length of stay 7.3 days [95% confidence interval (CI) 5.7–8.9]. In nine controlled studies there was no difference in mortality [risk ratio (RR) 0.89, 95% CI 0.67–1.19] by comparing pre-PERT with PERT era. When analysing patients with intermediate or high-risk class only, the effect estimate for mortality tended to be lower for patients treated in the PERT era compared to those treated in the pre-PERT era (RR 0.71, 95% CI 0.45–1.12). The use of advanced therapies was higher (RR 2.67, 95% CI 1.29–5.50) and the in-hospital stay shorter (mean difference − 1.6 days) in PERT era compared to pre-PERT era.

**Conclusions:**

PERT implementation led to greater use of advanced therapies and shorter in-hospital stay. Our meta-analysis did not show a survival benefit in patients with PE since PERT implementation. Large prospective studies are needed to further explore the impact of PERTs on clinical outcomes.

**Registration:**

Open Science Framework 10.17605/OSF.IO/SBFK9.

**Graphical abstract:**

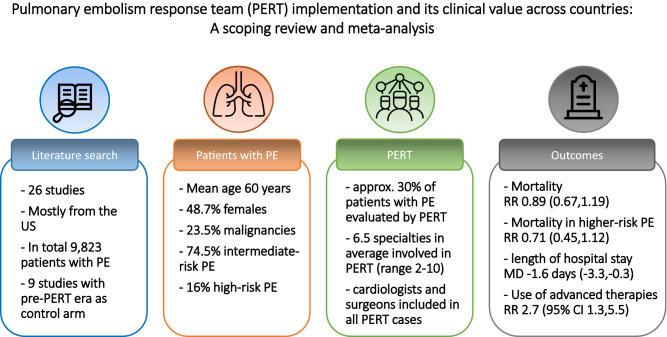

**Supplementary Information:**

The online version contains supplementary material available at 10.1007/s00392-022-02077-0.

## Introduction

Acute pulmonary embolism (PE) is one of the most frequent cardiovascular emergencies and is of particular clinical relevance due to its life-threatening potential in case of cardiorespiratory decompensation [1]. Patients with acute PE constitute a heterogeneous group of patients, and therefore, the 2019 European Society of Cardiology (ESC) Guidelines emphasise the importance of risk stratification to define appropriate management strategies [2]. Over the last decade, the array of treatment options for PE has rapidly expanded; especially advanced treatment options, such as catheter-directed treatment, are increasingly attracting attention in the management of acute PE [3]. However, the increasing variety and complexity in treatment options and the need for implementation of tailored strategies raise the importance of interdisciplinary communication and collaboration.

The “heart team” concept for multidisciplinary management of patients with challenging cardiovascular diseases is meanwhile established and is gaining increasing acceptance worldwide [4]. Originating from the same conceptual framework, multidisciplinary rapid-response teams for the management of “severe” PE, known as pulmonary embolism response teams (PERTs), could help to optimise treatment for acute PE [5]. Members of the PERT meet in real time to ensure rapid clinical decision making and may include, depending on the local resources and expertise, specialists from cardiology, radiology, pulmonology, haematology, anaesthesiology and cardiothoracic surgery [6]. Little is known about the general composition and clinical value of PERT in daily clinical practice. We, therefore, conducted the present scoping review and meta-analysis to investigate the composition of PERTs across different countries and determine the added clinical value since its implementation.

## Materials and methods

### Study objectives

The objectives of the present scoping review and meta-analysis were: (1) to identify the published evidence regarding the implementation of PERTs in acute PE treatment, (2) to clarify the key characteristics in the structure, function and operating procedures of PERTs worldwide, and (3) to identify knowledge gaps concerning PERTs. The present review was performed according to the PRISMA extension guidelines for scoping reviews [7]. The protocol for this study has been registered in the Open Science Framework (117605/OSF.IO/SBFK9).

### Data sources and searches

A systematic search of MEDLINE (via PubMed), the Cochrane Central Register of Controlled Trials (CENTRAL) and Web of Science was performed up to 10 January 2022. A search string was created for PubMed and modified accordingly for the other databases (Supplement 1). To complement our search, all references from selected studies were retrieved and manually reviewed according to the snowball effect. No language restrictions were set.

### Inclusion and exclusion criteria

We considered full-text, prospective and retrospective observational studies, which included patients with acute PE evaluated by a PERT. Both controlled and uncontrolled (single-group) studies were eligible. In controlled studies, the (historical) control group consisted of patients with acute PE who were treated before the implementation of a PERT. Eligible articles were designed to either describe the structure and function of PERTs and/or to investigate outcomes related to the implementation of a PERT. The main outcome was all-cause mortality (overall, in-hospital or 30-day mortality). Additional outcomes were the occurrence of bleeding (overall and major bleeding), 30-day rehospitalisation rates, length of hospitalisation, use of intensive care unit (ICU), length of stay in the ICU, use of advanced therapies [comprising systemic full- or half-dose thrombolysis, catheter-directed treatment (CDT) including catheter-directed thrombolysis or percutaneous thrombectomy, surgical thrombectomy and extracorporeal membranous oxygenation (ECMO)]; the insertion of a vena cava [IVC] filter was also evaluated. We excluded case reports and non-peer-reviewed articles.

### Study selection

Retrieved studies were imported into a reference management software (Mendeley version 1.19). After the removal of duplicated studies, two independent authors (IF, AM) at a first stage screened the titles and abstracts and at a second stage perused the full texts for eligible studies. A third author (LH) was consulted to resolve any discordance regarding eligibility of studies. All reasons for exclusion at the full-text study selection phase were reported.

### Data extraction and quality assessment

We created a predefined excel spreadsheet into which two authors (IF, AM) independently extracted data from eligible studies. A pilot test was performed before the formal initiation of data extraction to ensure coherence. Any disagreement was resolved by consensus. We extracted data regarding the study design of each study (country of corresponding author, academic setting or not, multicenter or not, prospective or retrospective design, presence of control group or not, and population inclusion and exclusion criteria), site-specific characteristics of the PERT (structure and number of specialties involved, setting of PERT activations, proportion of patients with acute PE for whom PERT was activated, and predictors of PERT activation), baseline characteristics of the population (mean age, female sex, mean body mass index, active malignancy, right ventricular dysfunction, proportion of patients with low, intermediate–low, intermediate–high and high risk acute PE) and outcomes (as described above). We performed a quality assessment of the eligible controlled studies using the ROBINS-I risk-of-bias tool for non-randomised studies of interventions [8].

### Statistical analysis

We performed a random effects model meta-analysis of controlled studies (DerSimonian and Laird method). The effect estimate was the risk ratio (RR) for binary outcomes and the mean difference for continuous outcomes, with corresponding 95% confidence intervals (CIs). Heterogeneity was assessed with the Cochran chi-square test and the I [2] statistic (values greater than 50% indicated high heterogeneity). A subgroup analysis was performed by only including patients with more severe PE (as defined per each study). Publication bias was assessed visually with the use of funnel plots. The analysis was performed using the meta package in R (version 3.6.3).

## Results

### Description of studies

**Fig. 1:**
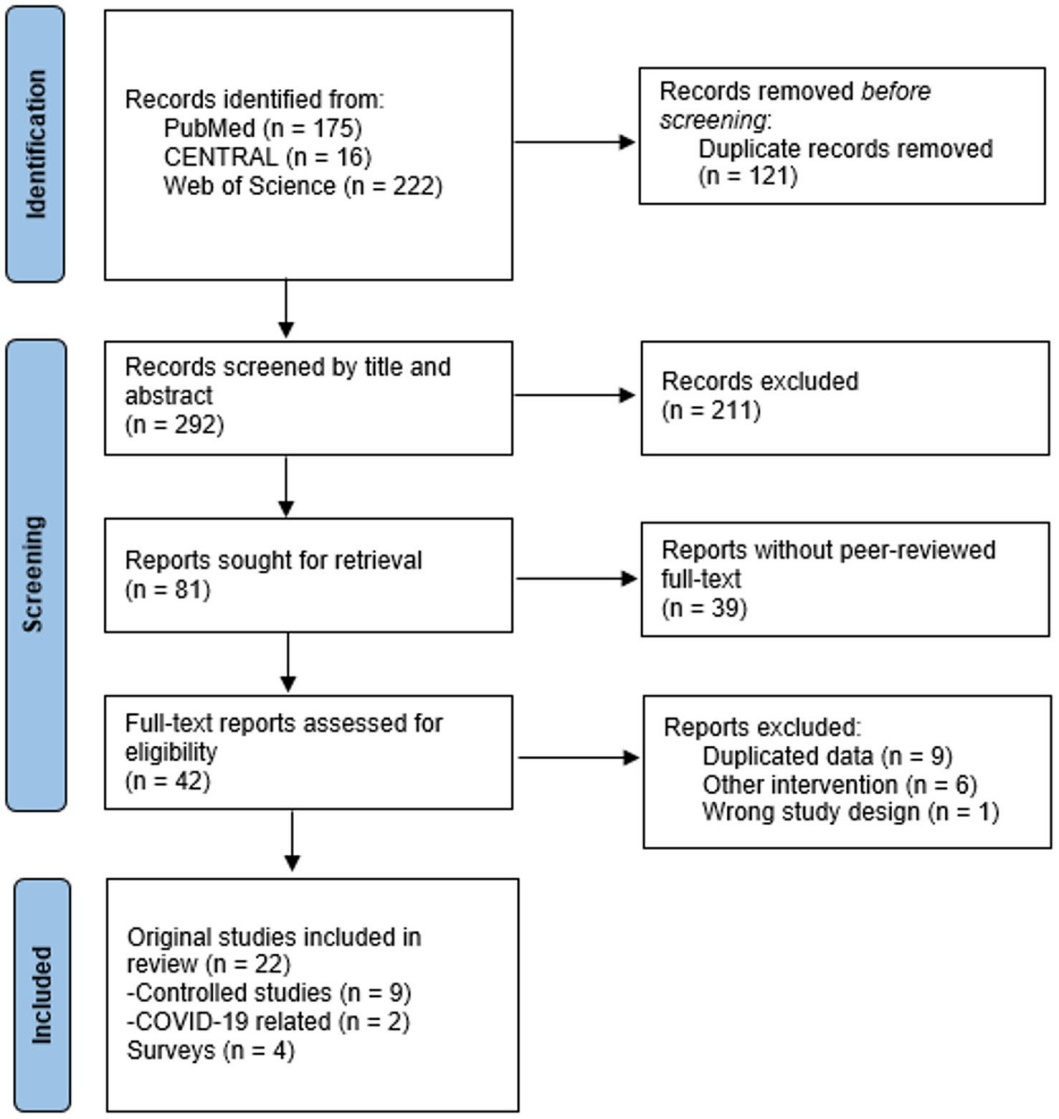
Flowchart of the study selection process

The search strategy resulted in the retrieval of 292 studies after removal of duplicates. Among them, we identified 26 (8.9%) reviews, 17 (5.8%) letters or editorials and 39 (13.4%) conference abstracts related to PERT implementation. After the complete study selection process, 26 studies were included in the final review and meta-analysis (Fig. 1). Of them, 22 were original research studies and four were physician surveys. Results of physician surveys were extracted separately and are shown in Table S1 [9–12]. The majority of the 22 original research studies originated from the US, with the exception of one from Canada, Poland and Singapore, respectively [13–35]. All studies, except for one, were performed in an academic setting and three were multicentre. A total of 9823 patients were included in the quantitative analysis. Characteristics of the included studies are presented in Table 1.

**Table 1:** Characteristics of the included studies

Study	Country	Population	Control	Number of patients	Age, years	Female, %	Cancer, %	RV Dysfunction, %	Risk groups, %
Annabathula et al. 2021 [14]	US	Acute PE (all-comers) exclusion criteria: no CTPA, no evaluation of the RV	Yes	530	I: 58.1 C: 59.5	I: 53 C: 58.4	I: 21.4 C: 23.9	I: 70.4 C: 61.5	NR
Araszkiewicz et al. 2021 [15]	Poland	All PERT activations	No	680	57.7	50.6	21.2	NR	Low: 22.8, Intermediate–low: 24.2, Intermediate–high: 42.9, High: 10.1
Carroll et al. 2020 [16]	US	Acute PE (all-comers)	Yes	2042	I: 63.6 C: 62.3	I: 53.9 C: 52.3	I: 29.2 C: 31.3	I: 36.1 C: 43.2	I: Low: 46.4, Intermediate: 49.8, High: 3.8 C: Low: 61.4, Intermediate: 33.8, High: 4.8
Chaudhury et al. 2019 [17]	US	Acute PE (all-comers) exclusion criteria: subsegmental PE, out-patient care	Yes	769	I: 57.2 C: 58.1	I: 47.9 C: 49.3	I: 31.9 C: 32.9	I: 28.9 C: 22.4	I: Low: 11.3, Intermediate and High: 88.7 C: Low: 15.7, Intermediate and High: 84.3
Deadmon et al. 2017 [18]^a^	US	All PERT activations	No	561	61.1	46.5	33.4	NR	Low: 15.7, Intermediate: 50.2, High: 34.2
Finn et al. 2021 [19]	US	PERT consultations before and after COVID-19	No	100	59.2	45	11	47.6	Intermediate and High: 65.7
Groth et al. 2021 [20]^b^	US	Acute PE, massive or submassive	Yes	573	I: 63.4 C: 63.2	I: 44.9 C: 48	NR	I: 79.9 C: 66	I: Intermediate–high: 79, High: 21 C: Intermediate–high: 74. High: 26
Jen et al. 2020 [21]	Singapore	Acute PE (all-comers)	Yes	321	I: 60.3 C: 61.1	I: 51.5 C: 51.9	I: 30.5 C: 26.6	NR	I: Low: 9, Intermediate: 79, High: 9.1 C: Low: 9.1, Intermediate: 82.5, High: 8.4
Kendall et al. 2018 [35]	US	PE patients with massive or submassive PE and evaluated by PERT	No	40	56	58	25	NR	Intermediate: 57, High: 43
Khaing et al. 2019 [23]	US	PE patients evaluated by PERT	No	52	56	55.8	19.2	NR	Low: 0, Intermediate: 94.2, High: 5.8
Kwok et al. 2021 [24]^c^	US	Acute PE (all-comers) before and after COVID-19	No	60				43.3	Low: 18.3, Intermediate: 76.6, High: 5
Melamed et al. 2020 [25]	US	Acute PE (all-comers)	Yes	728	I: 62.4 C: 62.4	I: 47.7 C: 52.4	I: 26.7 C: 20.5	NR	NR
Mortensen et al. 2021 [26]^a^	US	Acute PE transferred to the ED	No		NR	48.1	39	NR	Low: 56.9, Intermediate and High: 43.1
Myc et al. 2020 [27]	US	Acute PE (all-comers)	Yes	554	I: 61.9 C: 62	I: 48.1 C: 48	I: 36.3 C: 33	NR	I: Low: 35, Intermediate: 36.6, High: 28 C: Low: 30, Intermediate: 36,7, High: 33
Parikh et al. 2021 [36]	US	PERT activations	No	69	60.3	47.8	20.3	NR	Low: 20.3, Intermediate: 65.2, High: 14.5
Romano et al. 2020 [29]	Canada	PERT activations	No	128	63	42	32	NR	Low: 3.1, Intermediate: 85.2, High: 11.7
Rosovsky et al. 2018 [5]^a^	US	Acute PE, eligible only those who met the hospital's criteria for PERT activation	Yes	440	I: 61 C: 59	I: 47 C: 52	I: 17 C: 26	NR	I: Low: 19.3, Intermediate: 49.1, High: 31.6 C: Low: 36.8, Intermediate: 31.6, High: 31.6
Schultz et al. 2018 [17]^d^	US	PERT activations	No	416	61.2	50.2	26.7	55.5	Low: 18.8, Intermediate: 69, High: 12.3
Sista et al. 2018 [31]	US	PERT activations, massive or submassive	No	87	63.7	49.4	33.3	NR	Low: 0, Intermediate: 90.8, High: 9.2
Wiske et al. 2020 [32]^c^	US	PERT activations	No	179	59.9	47.4	30.3	33	Intermediate: 91.3, High: 8.7
Wright et al. 2021 [33]^b^	US	PERT activations, massive or submassive	Yes	368	I: 63.9 C: 63.2	I: 46 C: 48	I: 23 C: 28	I: 84 C: 66	I: Low: 0, Intermediate–low: 36.8, Intermediate–high: 46.8, High: 16.5 C: Low: 0, Intermediate–low: 45.3, Intermediate–high: 28.5 High: 26.3
Xenos et al. 2019 [34]	US	PERT activations	Yes	1069	I: 58.5 C: 56.6	I: 45.5 C: 51.4	NR	NR	Intermediate–high: 87, High: 13

^a^studies from the Massachusetts general hospital

^b^studies from the university of Rochester medical center/strong memorial

^c^studies for the university Langone New York

^d^multicenter study comprising several centers included in this review. All studies with duplicated data were not pooled together to avoid unit-of-analysis error

*C* control population (not evaluated by PERT), *CTPA* computed tomography pulmonary angiogram, *ED* emergency department, *I* intervention population (evaluated by PERT), *NR* not reported, *PE* pulmonary embolism, *PERT* pulmonary embolism response team, *RV* right ventricle, *VTE* venous thromboembolism

### Composition and operation of PERTs

**Fig. 2:**
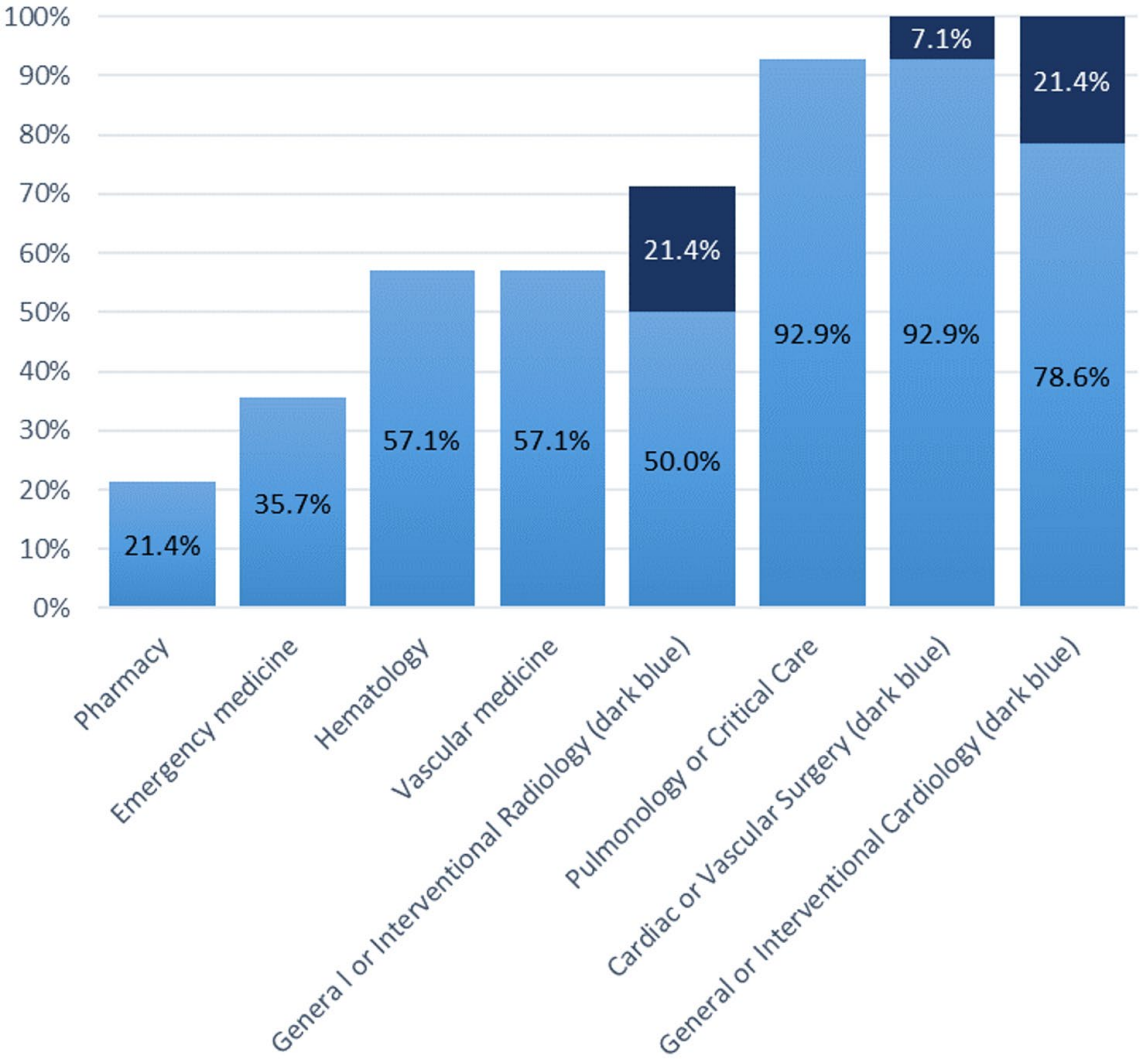
Participation rate of specialties in PERT across 13 original studies

Overall, 31.5% of patients with acute PE were, irrespectively of their risk class, evaluated by a PERT across 8 studies [14, 16, 17, 24–27, 36]. The median number of specialties involved in PERT across all included studies was 6.5 (range 2–10). Up to 11 different specialties were involved in PERTs. The participating rate of each specialty is presented in Fig. 2, [5, 15, 16, 18, 19, 21, 23, 27, 29, 31, 33–35]. A single pager number, a dedicated phone line, or an alert via the electronic medical system were the tools for PERT activation across studies. Reasons for PERT activation, as reported in two studies, were the patient’s clinical presentation (particularly the presence of tachycardia, hypotension and hypoxia), right ventricular dysfunction, history of prior VTE or thrombophilia, family history of VTE, or presence of malignancy or recent surgery [26, 36]. Referrals originated mostly by emergency departments (59.4%), followed by medical or surgery wards (29.1%), and ICU (9.9%). Patients evaluated by a PERT had a mean age of 60 years; among these 48.7% were females, and 23.5% suffered from malignancy. Right ventricular dysfunction was present in 55% of the patients. In total, 74.5% were classified as having intermediate-risk PE and 16% as high-risk PE.

### Quality assessment of included controlled studies

Nine controlled studies were assessed for risk of bias with the ROBINS-I tool; four of them were found to be of high risk of bias, while the rest of moderate risk of bias (Figure S1) [5, 14, 16, 17, 21, 25, 27, 33, 34]. A significant proportion of bias was identified in the bias due to confounding (44% of studies with high risk of bias). The “traffic light” plot for the risk of bias in each category of the individual studies is shown in Figure S2.

### Mortality regarding intermediate- and high-risk patients according to PERT implementation

Overall, 11 studies (*n* = 1532 patients) reported outcomes for the subgroup of intermediate- and high-risk patients who were evaluated by a PERT [5, 14, 16, 21, 23, 29, 31–35]. In this subgroup of patients, the pooled mortality rate reached 10% [177/1532 patients (95% CI 8–13%)], the pooled bleeding rate 9% [119/1221 patients (95% CI 7% to 12%)] and the mean length of stay was 7.3 days (95% CI 5.7–8.9 days). The use of any advanced therapy was high (393/1532 patients, 30%) and, in particular, 6% for systemic thrombolysis (89/1405 patients), 22% for CDT (266/1532 patients), 2% for surgical thrombectomy (21/986 patients) and 3% for ECMO (34/1018 patients); an IVC filter was inserted in 15% of patients (79/543 patients).

### Clinical course of patients in the pre-PERT and PERT era

**Fig. 3:**
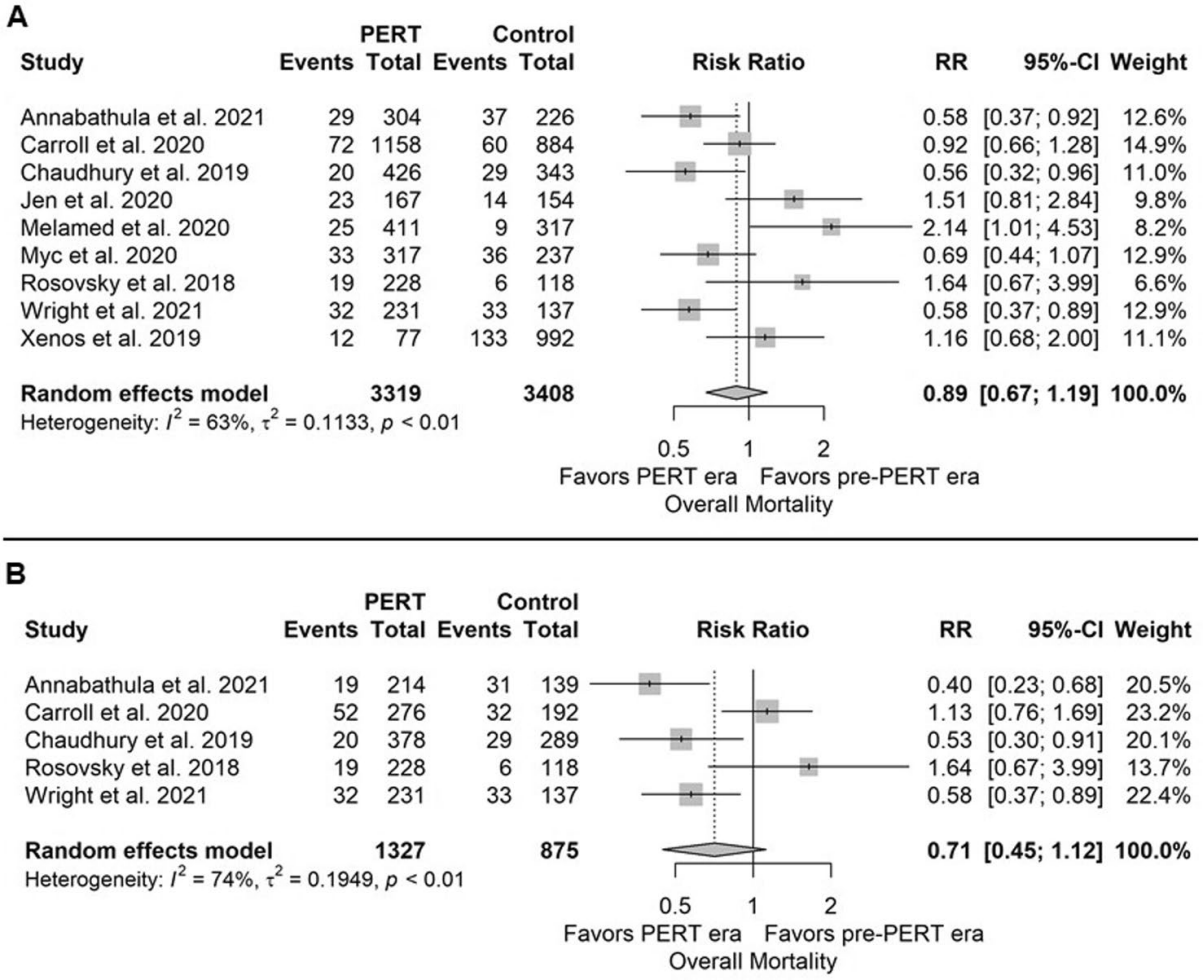
Risk ratio regarding risk of mortality in patients across all risk groups (**A**) and across intermediate and high-risk PE groups (**B**)

After pooling nine controlled studies, our meta-analysis comprised a total of 6,821 patients [5, 14, 16, 17, 21, 25, 27, 33, 34]. No difference in mortality was observed between the pre-PERT and PERT era when taking all risk classes into consideration (RR 0.89, 95% CI 0.67 to 1.19, Fig. 3A). When analysing patients with intermediate or high-risk PE only, the effect estimate for mortality were lower for patients treated in the PERT era compared to patients treated in the pre-PERT era; however, no statistical significance was achieved (RR 0.71, 95% CI 0.45–1.12, Fig. 3B). The heterogeneity among studies was high (*I*^2^ = 63%, *p* < 0.01). The funnel plot indicated that studies with a larger number of patients showed a favourable effect of PERT implementation on mortality, whereas smaller studies were more likely to report a RR > 1.0 (Figure S3).

No differences in the 30-day readmission, bleeding (major and overall), and ICU admission rates were found in the whole population and in the subgroup of patients with intermediate or high-risk PE. However, the total length of hospital stay was lower in the PERT era compared to the pre-PERT era (MD − 1.61 days, 95% CI − 3.21 – − 0.02 days); this also applied to the length of stay in the ICU (MD -1.79 days, 95% CI − 3.29 – − 0.28 days). Heterogeneity was high (> 90%, *p* < 0.01) for both continuous outcomes.

Use of advanced therapies (pooled rate) was more frequent in the PERT era compared to the pre-PERT era (RR 2.67, 95% CI 1.29–5.50, *I*^2^ = 95%, *p* < 0.01). For example, rates were higher for systemic thrombolysis [181/3242 (5.6%) in the PERT era vs. 79/2510 (3.1%) in the pre-PERT era; RR 1.70 (95% CI 0.73–3.98)] and CDT [214/3319 (6.4%) vs. 104/3502 (3.0%); RR 3.30 (95% CI 1.28–8.48)], but not for surgical thrombectomy [22/2527 (0.9%) vs. 15/1967 (0.8%); RR 0.87 (95% CI 0.29–2.62)], or ECMO [31/2513 (1.2%) vs. 34/2819 (1.3%); RR 1.76 (95% CI 0.72–4.32)]. Use of IVC filters was less frequent in the PERT compared to the pre-PERT era [205/2132 (9.6%) vs. 233/1601 (14.6%); RR 0.67 (95% CI 0.56–0.80)].

## Discussion

Acute PE is the most severe clinical manifestation of VTE; in case of haemodynamic instability, the short-term mortality rate ranges from 16 to 46% in patients with shock and from 52 to 84% in patients with cardiac arrest [1, 37]. The rationale behind the implementation of multidisciplinary PERT is to (1) improve the management of patients with life-threatening PE and (2) prevent cardiopulmonary arrest and death [38, 39]. Little is known about the effect of PERT implementation on clinical outcomes across different countries. To our knowledge, this is the first scoping review and meta-analysis addressing outcomes of patients with acute PE based on the availability of a PERT for clinical management and decision making.

The results of the present analysis indicate that the implementation of PERTs is a concept still predominantly implemented in the US. Even though the concept of PERT teams has been endorsed by the 2019 ESC Guidelines, only one study originated in Europe, notably in Poland, and reported on the composition and function of PERTs [15]. The rationale behind the implementation and structure of PERTs is based on the “heart team” concept, which facilitates patient management with a consensus opinion of different specialists and leads to improved organisation of teams and utilisation of resources [4]. In most cases, the number of PERT activations increased early after the implementation of PERT, suggesting both a learning curve and growing motivation of teams involved [17, 23]. In response to increasing treatment options for acute PE, each member of a PERT contributes with their own perspective based on their clinical and/or procedural expertise. A consensus recommendation by the National PERT Consortium™, established in 2015 in the US, suggests the composition of PERTs from specialists in the fields of cardiac surgery, cardiac imaging, interventional and non-interventional cardiology, critical care, emergency medicine, haematology, clinical pharmacy, pulmonary, diagnostic and interventional radiology, vascular medicine, and vascular surgery [40]. In our study, cardiologists or cardiac/vascular surgeons were included in all PERT activations, followed by pulmonologists or critical care physicians (92.9%) and radiologists (71.4%). Our results are in line with previous studies, in which substantial variations between institutions in terms of organisation, frequency of PERT activation and composition of PERTs were reported [41, 42]. The members of a PERT team should be adapted based on organisational and availability patterns in each institution [40]. However, as a general rule, a PERT is expected to involve at least one medical specialist (for example, a cardiologist, pulmonologist, haematologist, vascular specialist or internist), an interventions specialist (such as an interventional cardiologist or radiologist), and (wherever available) a cardiac or vascular surgeon.

The direct impact of PERTs on patient outcomes remains uncertain to date, since no direct prospective comparisons have been performed. In a retrospective singe-centre study, in which 769 patients with acute PE were divided in two groups corresponding to PE management in the pre-PERT and PERT era, all-cause 30-day mortality rate was significantly lower in patients treated in the PERT compared to the pre-PERT era (8.5% vs. 4.7%; *p* = 0.034) [17]. In the present analysis, we found no differences in mortality between patients managed in the pre-PERT vs. the PERT era when taking all patients, regardless of PE risk class, into consideration. However, since the PERT concept aims to standardise the care of patients with *severe* PE, comparison of outcomes in low-risk patients is of limited clinical relevance in this context [43]. Even if some original studies included patients with acute low-risk PE evaluated by PERT [41], we focused on predictors used for PERT activation. Except for elevated troponin levels, also other parameters of right ventricular decompensation, such as hypoxia, high respiratory rate or mild hypotension, played a decisive role in the activation of PERT teams underlining the importance of PERTs particular for patients with severe PE [28]. In fact, only 3 out of 10 all-comers with PE are evaluated by a PERT [28, 44]; these are the patients for whom complex management decisions are needed. After including in the analysis only high-risk or intermediate-risk patients with PE, the effect estimate for mortality were lower, but not statistical significant for patients treated in the PERT era compared to patients treated in the pre-PERT era, likely due to small patient numbers.

Regardless of the risk class, length of the general and the ICU in-hospital stay was lower in the PERT era as compared to the pre-PERT era. Although the length of hospital stay may be considered a rather subjective outcome as the criteria for discharge were likely different across sites, our results suggest that the implementation of PERT in an institution may provide confidence for earlier discharge in stabilised patients with acute PE. Besides, among patients with intermediate risk PE, patients who undergo invasive therapies have been shown to have a shorter length of stay in the hospital [45]. The cost efficiency of the administrative costs for setting up a PERT vs. the expected cost reduction resulting from reduced hospitalisation duration and provision of more reasonable use of advanced treatment modalities remains to be investigated.

Treatment options for patients with acute PE have expanded [46]; thus a PERT should help to justify the optimal treatment approach in selected patients [17, 44]. A recent single-centre trend analysis demonstrated that PERT implementation resulted in more than a tenfold increase in the frequency of CDT use as compared to the period before the introduction of PERT [5]. Furthermore, Carroll et al. described comparable findings for increase in the use of CDT after PERT implementation [16]. Our meta-analysis indicates that PERT led to an approximately 2.5-fold increase in the use of advanced therapies, mostly driven by an increase in the use of CDT; this implies that the confidence of physicians in the use of advanced therapies is increasing. Except for CTD, systemic thrombolysis also was used more frequently after PERT implementation. Our analysis further showed that the increased use of advanced therapies in the PERT era does not appear to be accompanied by an increase in the rate of major bleeding. In this context, it needs to be mentioned that our analysis was not powered to show statistically significant differences in mortality rates between patients treated in the pre- and post-PERT era. Major randomised controlled trials, aiming to clinically validate catheter-directed modalities for intermediate-risk and high-risk PE, are currently ongoing [3] or are being planned. If positive, their results can be expected to further promote implementation of PERTs in the future.

Our study has some limitations. First, several of the included studies were post-hoc analyses of existing cohorts, hence the results are purely observational and no cause-and-effect relationship can be established. Second, conclusions regarding clinical outcomes cannot be made due to the fact that the numerical analysis was only explorative. Third, not all controlled studies reported the outcomes of subgroups with intermediate- and high-risk PE separately, which reduced the power of the numerical analysis. Finally, the definition of intermediate- and high-risk PE was not standardised across studies, contributing to heterogeneity in the analysis.

In conclusion, in our study we were able to analyse the association between PERT-based management and clinical outcomes in 9823 patients with acute PE. Our meta-analysis did not demonstrate an effect estimate on mortality in patients with intermediate- or high-risk PE of PERT implementation compared to the pre-PERT era. However, PERT implementation was associated with increasing use of advanced therapies and lower length of in-hospital stay. Our study should be considered hypothesis generating; large prospective observational studies are needed to further explore the impact of PERT teams on clinical outcomes and mortality in patients with acute PE.

## Supplementary Information

Below is the link to the electronic supplementary material.

Supplementary file1 (DOCX 319 KB)

## Data Availability

The data underlying this article are available in the article and in its online supplementary material.

## References

[1] Keller K, Hobohm L, Ebner M, Kresoja KP, Munzel T, Konstantinides SV, Lankeit M (2020). Trends in thrombolytic treatment and outcomes of acute pulmonary embolism in Germany. Eur Heart J.

[2] Konstantinides SV, Meyer G, Becattini C, Bueno H, Geersing GJ, Harjola VP, Huisman MV, Humbert M, Jennings CS, Jimenez D, Kucher N, Lang IM, Lankeit M, Lorusso R, Mazzolai L, Meneveau N, Ni Ainle F, Prandoni P, Pruszczyk P, Righini M, Torbicki A, Van Belle E, Zamorano JL, and Group ESCSD (2020). ESC Guidelines for the diagnosis and management of acute pulmonary embolism developed in collaboration with the European Respiratory Society (ERS). Eur Heart J.

[3] Hobohm L, Keller K, Munzel T, Gori T, Konstantinides SV (2020). EkoSonic(R) endovascular system and other catheter-directed treatment reperfusion strategies for acute pulmonary embolism: overview of efficacy and safety outcomes. Expert Rev Med Devices.

[4] Holmes DR, Rich JB, Zoghbi WA, Mack MJ (2013). The heart team of cardiovascular care. J Am Coll Cardiol.

[5] Rosovsky R, Chang Y, Rosenfield K, Channick R, Jaff MR, Weinberg I, Sundt T, Witkin A, Rodriguez-Lopez J, Parry BA, Harshbarger S, Hariharan P, Kabrhel C (2019). Changes in treatment and outcomes after creation of a pulmonary embolism response team (PERT), a 10-year analysis. J Thromb Thrombolysis.

[6] Dudzinski DM, Piazza G (2016). Multidisciplinary pulmonary embolism response teams. Circulation.

[7] Tricco AC, Lillie E, Zarin W, O'Brien KK, Colquhoun H, Levac D, Moher D, Peters MDJ, Horsley T, Weeks L, Hempel S, Akl EA, Chang C, McGowan J, Stewart L, Hartling L, Aldcroft A, Wilson MG, Garritty C, Lewin S, Godfrey CM, Macdonald MT, Langlois EV, Soares-Weiser K, Moriarty J, Clifford T, Tuncalp O, Straus SE (2018). PRISMA extension for scoping reviews (PRISMA-ScR): checklist and explanation. Ann Intern Med.

[8] Sterne JA, Hernan MA, Reeves BC, Savovic J, Berkman ND, Viswanathan M, Henry D, Altman DG, Ansari MT, Boutron I, Carpenter JR, Chan AW, Churchill R, Deeks JJ, Hrobjartsson A, Kirkham J, Juni P, Loke YK, Pigott TD, Ramsay CR, Regidor D, Rothstein HR, Sandhu L, Santaguida PL, Schunemann HJ, Shea B, Shrier I, Tugwell P, Turner L, Valentine JC, Waddington H, Waters E, Wells GA, Whiting PF, Higgins JP (2016). ROBINS-I: a tool for assessing risk of bias in non-randomised studies of interventions. BMJ.

[9] Wang X, Ji QW, Rosenfield K, Tapson V, Nie SP (2021). Multidisciplinary pulmonary embolism response team in China: a nationwide survey. Respirology.

[10] Todoran TM, Giri J, Barnes GD, Rosovsky RP, Chang YC, Jaff MR, Rosenfield K, Kabrhel C, and Consortium P (2018). Treatment of submassive and massive pulmonary embolism: a clinical practice survey from the second annual meeting of the pulmonary embolism response team consortium. J Thromb Thrombol.

[11] Brailovsky Y, Kunchakarra S, Lakhter V, Barnes G, Masic D, Mancl E, Porcaro K, Bechara CF, Lopez JJ, Simpson K, Mathew V, Fareed J, Darki A (2020). Pulmonary embolism response team implementation improves awareness and education among the house staff and faculty. J Thromb Thrombolysis.

[12] Barnes G, Giri J, Courtney DM, Naydenov S, Wood T, Rosovsky R, Rosenfield K, Kabrhel C (1995). Nuts and bolts of running a pulmonary embolism response team: results from an organizational survey of the National PERT™ Consortium members. Hosp Pract.

[13] [Recommendations of the Polish Gynecological Society expert panel on the use of Detramax in pregnancy]. Ginekol Pol 2015;86:962–5. PMID: 26995949 26995949

[14] Annabathula R, Dugan A, Bhalla V, Davis GA, Smyth SS, Gupta VA (2021). Value-based assessment of implementing a pulmonary embolism response team (PERT). J Thromb Thrombolysis.

[15] Araszkiewicz A, Kurzyna M, Kopec G, Slawek-Szmyt S, Wrona K, Stepniewski J, Jankiewicz S, Pietrasik A, Machowski M, Darocha S, Mularek-Kubzdela T, Torbicki A, Pruszczyk P, Roik M (2021). Pulmonary embolism response team: a multidisciplinary approach to pulmonary embolism treatment. Polish PERT Ini Rep Kardiol Polska.

[16] Carroll BJ, Beyer SE, Mehegan T, Dicks A, Pribish A, Locke A, Godishala A, Soriano K, Kanduri J, Sack K, Raber I, Wiest C, Balachandran I, Marcus M, Chu L, Hayes MM, Weinstein JL, Bauer KA, Secemsky EA, Pinto DS (2020). Changes in care for acute pulmonary embolism through a multidisciplinary pulmonary embolism response team. Am J Med.

[17] Chaudhury P, Gadre S, Schneider E, Renapurkar R, Gomes M, Haddadin I, Heresi G, Tong MZY, Bartholomew JR (2019). Impact of multidisciplinary pulmonary embolism response team availability on management and outcomes. Am J Cardiol.

[18] Deadmon EK, Giordano NJ, Rosenfield K, Rosovsky R, Parry BA, Al-Bawardy RF, Chang YC, Kabrhel C (2017). Comparison of emergency department patients to inpatients receiving a pulmonary embolism response team (PERT) activation. Acad Emerg Med.

[19] Finn MT, Gogia S, Ingrassia JJ, Cohen M, Madhavan MV, Nouri SN, Brailovsky Y, Masoumi A, Fried JA, Uriel N, Agerstrand CI, Eisenberger A, Einstein AJ, Brodie D, Rosenzweig EB, Leon MB, Takeda K, Pucillo A, Green P, Kirtane AJ, Parikh SA, Sethi SS (2021). Pulmonary embolism response team utilization during the COVID-19 pandemic. Vasc Med.

[20] Groth CM, Acquisto NM, Wright C, Marinescu M, McNitt S, Goldenberg I and Cameron SJ Pharmacists as members of an interdisciplinary pulmonary embolism response team J Am Coll Clin Pharm. PMID: 35813573 10.1002/jac5.1569 PMC9269076 35813573

[21] Jen WY, Kristanto W, Teo L, Phua J, Yip HS, MacLaren G, Teoh K, Sim TB, Loh J, Ong CC, Chee YL, Kojodjojo P (2020). Assessing the impact of a pulmonary embolism response team and treatment protocol on patients presenting with acute pulmonary embolism. Heart Lung Circul.

[22] Kabrhel C, Rosovsky R, Channick R, Jaff MR, Weinberg I, Sundt T, Dudzinski DM, Rodriguez-Lopez J, Parry BA, Harshbarger S, Chang YC, Rosenfield K (2016). A multidisciplinary pulmonary embolism response team initial 30-month experience with a novel approach to delivery of care to patients with submassive and massive pulmonary embolism. Chest.

[23] Khaing P, Paruchuri A, Eisenbrey JR, Merli GJ, Gonsalves CF, West FM, Awsare BK (1995). First year experience of a pulmonary embolism response team with comparisons of outcomes between catheter directed therapy versus standard anticoagulation. Hosp Pract.

[24] Kwok B, Brosnahan SB, Amoroso NE, Goldenberg RM, Heyman B, Horowitz JM, Jamin C, Sista AK, Smith DE, Yuriditsky E, Maldonado TS (2021). Pulmonary embolism response team activation during the COVID-19 pandemic in a New York city academic hospital: a retrospective cohort analysis. J Thromb Thrombolysis.

[25] Melamed R, St Hill CA, Engstrom BI, Tierney DM, Smith CS, Agboto VK, Weise BE, Eckman PM and Skeik N (2020) Effects of a consensus-based pulmonary embolism treatment algorithm and response team on treatment modality choices, outcomes, and complications Clin Appl Thromb-Hemost 26. PMID: 32539524 10.1177/1076029620928420 PMC7427027 32539524

[26] Mortensen CS, Kramer A, Schultz JG, Giordano N, Zheng H, Andersen A, Nielsen-Kudsk JE and Kabrhel C Predicting factors for pulmonary embolism response team activation in a general pulmonary embolism population J Thromb Thrombolysis. PMID: 34370168 10.1007/s11239-021-02533-0 34370168

[27] Myc LA, Solanki JN, Barros AJ, Nuradin N, Nevulis MG, Earasi K, Richardson ED, Tsutsui SC, Enfield KB, Teman NR, Haskal ZJ, Mazimba S, Kennedy JLW, Mihalek AD, Sharma AM and Kadl A (2020) Adoption of a dedicated multidisciplinary team is associated with improved survival in acute pulmonary embolism Resp Res 21. PMID: 32571318 10.1186/s12931-020-01422-z PMC7310489 32571318

[28] Parikh M, Chahine NM, Hammad TA, Tefera L, Li J, Carman T, Schilz R, Shishehbor MH (2021). Predictors and potential advantages of PERT and advanced therapy use in acute pulmonary embolism. Catheter Cardiovasc Interv.

[29] Romano KR, Cory JM, Ronco JJ, Legiehn GM, Bone JN, Finlayson GN (2020). Vancouver general hospital pulmonary embolism response team (VGH PERT): initial three-year experience. Canadian J Anesth-J Canadien D Anesth.

[30] Rosovsky R, Chang YC, Rosenfield K, Channick R, Jaff MR, Weinberg I, Sundt T, Witkin A, Rodriguez-Lopez J, Parry BA, Harshbarger S, Hariharan P, Kabrhel C (2019). Changes in treatment and outcomes after creation of a pulmonary embolism response team (PERT), a 10-year analysis (vol 47, pg 31, 2019). J Thromb Thrombolysis.

[31] Sista AK, Friedman OA, Dou E, Denvir B, Askin G, Stern J, Estes J, Salemi A, Winokur RS, Horowitz JM (2018). A pulmonary embolism response team's initial 20 month experience treating 87 patients with submassive and massive pulmonary embolism. Vasc Med.

[32] Wiske CP, Shen C, Amoroso N, Brosnahan SB, Goldenberg R, Horowitz J, Jamin C, Sista AK, Smith D, Maldonado TS (2020). Evaluating time to treatment and in-hospital outcomes of pulmonary embolism response teams. J Vasc Surg-Venous Lymph Disord.

[33] Wright C, Goldenberg I, Schleede S, McNitt S, Gosev I, Elbadawi A, Pietropaoli A, Barrus B, Chen YL, Mazzillo J, Acquisto NM, Van Galen J, Hamer A, Marinescu M, Delehanty J, Cameron SJ (2021). Effect of a multidisciplinary pulmonary embolism response team on patient mortality. Am J Cardiol.

[34] Xenos ES, Davis GA, He Q, Green A, Smyth SS (2019). The implementation of a pulmonary embolism response team in the management of intermediate- or high-risk pulmonary embolism. J Vasc Surg-Ven Lymph Disord.

[35] Kendall MR, Swadron S, Clavijo LC, Mehra AK, Hindoyan A, Matthews RV, Shavelle DM (2018). Us of the STEMI team for treatment of patients with pulmonary embolism: a pilot study. J Invas Cardiol.

[36] Parikh M, Chahine NM, Hammad TA, Tefera L, Li J, Carman T, Schilz R, Shishehbor MH (2021). Predictors and potential advantages of PERT and advanced therapy use in acute pulmonary embolism. Catheter Cardiovasc Intervent.

[37] Hobohm L, Sagoschen I, Habertheuer A, Barco S, Valerio L, Wild J, Schmidt FP, Gori T, Munzel T, Konstantinides S and Keller K (2021) Clinical use and outcome of extracorporeal membrane oxygenation in patients with pulmonary embolism Resuscitation. PMID: 34653550 10.1016/j.resuscitation.2021.10.007 34653550

[38] Winters BD, Weaver SJ, Pfoh ER, Yang T, Pham JC, Dy SM (2013). Rapid-response systems as a patient safety strategy: a systematic review. Ann Intern Med.

[39] Mahar JH, Haddadin I, Sadana D, Gadre A, Evans N, Hornacek D, Mahlay NF, Gomes M, Joseph D, Serhal M, Tong MZ, Bauer SR, Militello M, Silver B, Shishehbor M, Bartholomew JR, Heresi GA (2018). A pulmonary embolism response team (PERT) approach: initial experience from the Cleveland clinic. J Thromb Thrombolysis.

[40] Rivera-Lebron B, McDaniel M, Ahrar K, Alrifai A, Dudzinski DM, Fanola C, Blais D, Janicke D, Melamed R, Mohrien K, Rozycki E, Ross CB, Klein AJ, Rali P, Teman NR, Yarboro L, Ichinose E, Sharma AM, Bartos JA, Elder M, Keeling B, Palevsky H, Naydenov S, Sen P, Amoroso N, Rodriguez-Lopez JM, Davis GA, Rosovsky R, Rosenfield K, Kabrhel C, Horowitz J, Giri JS, Tapson V, Channick R, and Consortium P (2019). Diagnosis, treatment and follow up of acute pulmonary embolism: consensus practice from the PERT Consortium. Clin Appl Thromb Hemost.

[41] Schultz J, Giordano N, Zheng H, Parry BA, Barnes GD, Heresi GA, Jaber W, Wood T, Todoran T, Courtney DM, Naydenov S, Khandhar S, Green P and Kabrhel C (2019) EXPRESS: a multidisciplinary pulmonary embolism response team (PERT): experience from a national multicenter consortium Pulm Circ 2045894018824563. PMID: 30632901 10.1177/2045894018824563 PMC6690111 30632901

[42] Barnes G, Giri J, Courtney DM, Naydenov S, Wood T, Rosovsky R, Rosenfield K, Kabrhel C, National PCRC (1995). Nuts and bolts of running a pulmonary embolism response team: results from an organizational survey of the national PERT consortium members. Hosp Pract.

[43] Brailovsky Y, Lakhter V (2021). pulmonary embolism response team: additional call burden or a valuable learning opportunity?. J Am Coll Cardiol.

[44] Lacey MJ, Hammad TA, Parikh M, Tefera L, Sharma P, Kahl R, Zemko A, Li J, Carman T, Schilz R, Shishehbor MH (2021). Prospective experience of pulmonary embolism management and outcomes. J Invasive Cardiol.

[45] Sullivan AE, Holder T, Truong T, Green CL, Sofela O, Dahhan T, Granger CB, Jones WS and Patel MR (2020) Use of hospital resources in the care of patients with intermediate risk pulmonary embolism Eur Heart J Acute Cardiovasc Care. PMID: 33609111 10.1177/2048872620921601 33242980

[46] Hobohm L, Schmidt FP, Gori T, Schmidtmann I, Barco S, Munzel T, Lankeit M, Konstantinides SV, Keller K (2021). In-hospital outcomes of catheter-directed thrombolysis in patients with pulmonary embolism. Eur Heart J Acute Cardiovasc Care.

