# Supplementary material for: Pulmonary embolism response team (PERT) implementation and its clinical value across countries: a scoping review and meta-analysis
Source: Clin Res Cardiol. 2022 Aug 17;112(10):1351–61. doi: 10.1007/s00392-022-02077-0 (PMC9383680; doi:10.1007/s00392-022-02077-0)
Supplement: Supplementary file 1 — Supplementary file1 (DOCX 319 KB) [file 392_2022_2077_MOESM1_ESM.docx]

**Supplementary Material**

**Supplement 1 – Search strategy**

PubMed (accessed 10/01/2022)

((pulmonary embolism) AND ((response) AND (team))) OR (pulmonary embolism response teams)

(("pulmonary embolism"[MeSH Terms] OR ("pulmonary"[All Fields] AND "embolism"[All Fields]) OR "pulmonary embolism"[All Fields]) AND (("response"[All Fields] OR "responses"[All Fields] OR "responsive"[All Fields] OR "responsiveness"[All Fields] OR "responsivenesses"[All Fields] OR "responsives"[All Fields] OR "responsivities"[All Fields] OR "responsivity"[All Fields]) AND "team"[All Fields])) OR (("pulmonary embolism"[MeSH Terms] OR ("pulmonary"[All Fields] AND "embolism"[All Fields]) OR "pulmonary embolism"[All Fields]) AND ("response"[All Fields] OR "responses"[All Fields] OR "responsive"[All Fields] OR "responsiveness"[All Fields] OR "responsivenesses"[All Fields] OR "responsives"[All Fields] OR "responsivities"[All Fields] OR "responsivity"[All Fields]) AND ("team s"[All Fields] OR "teamed"[All Fields] OR "teaming"[All Fields] OR "teamness"[All Fields] OR "teams"[All Fields]))

**Supplement 2 – Physician survey results**

| Survey | Description and scope of survey | Timeframe | Response rate | Center description | Specialty involvement in PERT | Activation of PERT | Use of advanced therapies | Other survey results |
| --- | --- | --- | --- | --- | --- | --- | --- | --- |
| Barnes et al. 2017[^12^](#_ENREF_12) | Survey among participating centers to the PERT Consortium™ in order to describe the function of PERT. | April 2016 – June 2016 | 79.5% of centers | Hospitals with 300-499 (11%), 500-749 (42%), 750-999 (32%), and ≥1000  (16%) hospital beds | Median 4 (IQR 3-6) specialties involved. 0.457). Pulmonary/critical care and at least one catheter-based specialty (interventional cardiology, interventional radiology, vascular surgery,  or interventional vascular medicine) were involved at all respondent institutions. | 42% used a dedicated PERT service for activation. Appropriateness for activation includes criteria for sub-massive or massive PE. | Availability of systemic thrombolysis (100%), inferior vena cava filter (100%), catheter-based therapy or catheter-based thrombectomy or surgical embolectomy (89%), extracorporeal membrane oxygenation (68%). | Follow-up for patients with PERT consultation was conducted in dedicated PERT clinic (47%) or personal clinic of a PERT physician (79%). |
| Brailovsky et al. 2020[^11^](#_ENREF_11) | Survey among physicians of a tertiary care center in order to assess the impact of PERT implementation on house staff education. | August 2017 – June 2018 | 12.5%-13.2% of individuals | N/A | N/A | N/A | Self-reported  comfort with explaining all available treatment  modalities to patients increased from 49.1 to 67.9% | Targeted educational  efforts increased the awareness of the PERT  from 72.2 to 92.6% (p < 0.01) and led to  a significant increase in PERT activations. (6.3 to 14.3 activations  per month, p < 0.01). Accurate clinical risk stratification of  acute PE increased from 60.2% to 73.8% (p = 0.03). |
| Todoran et al. 2018[^10^](#_ENREF_10) | Survey among 100 physicians participating to the PERT Consortium™ in order to better record the risk stratification and treatment of patients with PE within the PERT. | June 2016 | 100% | 70% academic centers, 66% hospitals with ≥500 beds | Interventional cardiology (27%), interventional radiology (20%),  critical care (17%) and vascular medicine (15%). | N/A | Treatment of choice when using a treatment other than anticoagulation: catheter-directed thrombolysis  (73%), full-dose systemic thrombolysis (12%), other non-thrombolytic approach (10%), half-dose  systemic thrombolysis (5%), other thrombolytic  approach (1%). | N/A |
| Wang et al. 2021[^9^](#_ENREF_9) | Nationwide survey of the China Consortium of PERT and the Pulmonary Vascular Disease Group of Chinese Society of Cardiology to record the current status of PERT practice in China. | September 2019 | 91.8% |  | 88% with ≥5 specialties.  Specialties more commonly involved were general cardiology  (96%), emergency medicine (82%) and pulmonary/critical care (82%) | 96% used telephone alerts and 37% a Wechat software to activate the PERT. Appropriate activation included high-risk (89%) and intermediate-high risk (89%) PE. | Availability: systemic thrombolysis (93%), inferior vena  cava filter placement (96%), catheter-based therapy (82%), surgical embolectomy (61%) and  extracorporeal  membrane  oxygenation  (86%). | N/A |

PE: pulmonary embolism; PERT: pulmonary embolism response team

**Supplement 3 – Quality assessment**

**Figure S1.** Overall risk of bias across included controlled studies


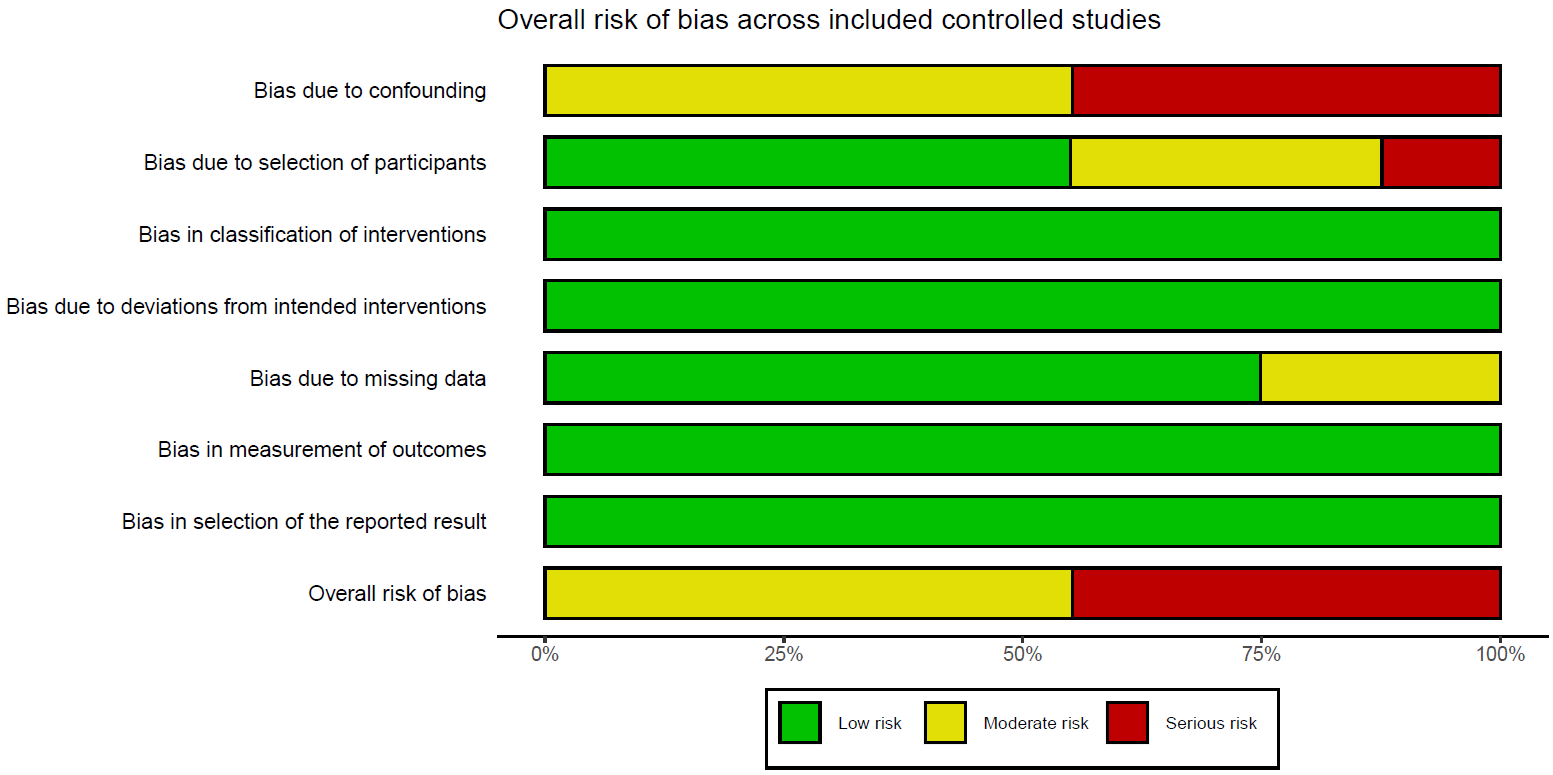


**Figure S2.** Traffic-light plot of individual included controlled studies


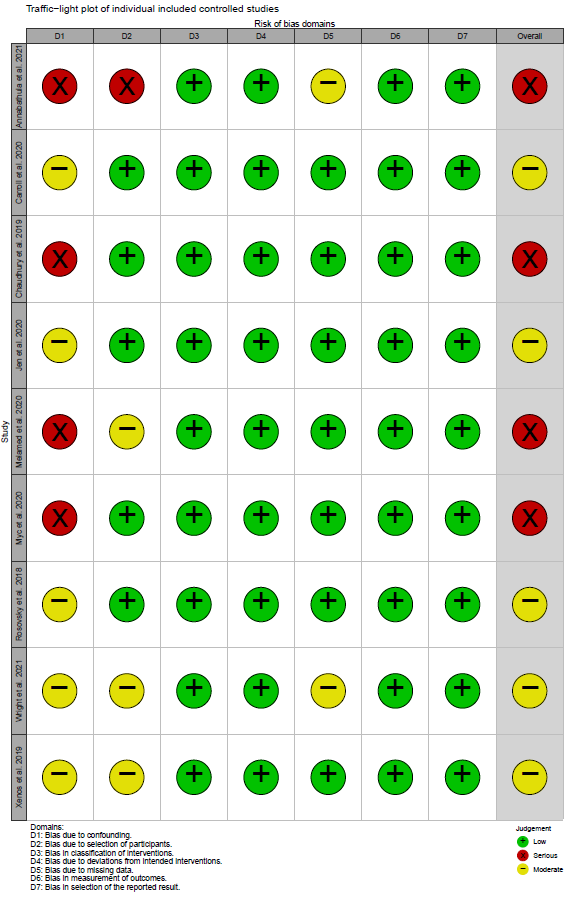


**Supplement 4 – Publication bias – Funnel plot**

**Figure S3.** Funnel plot of controlled studies with mortality outcome

**
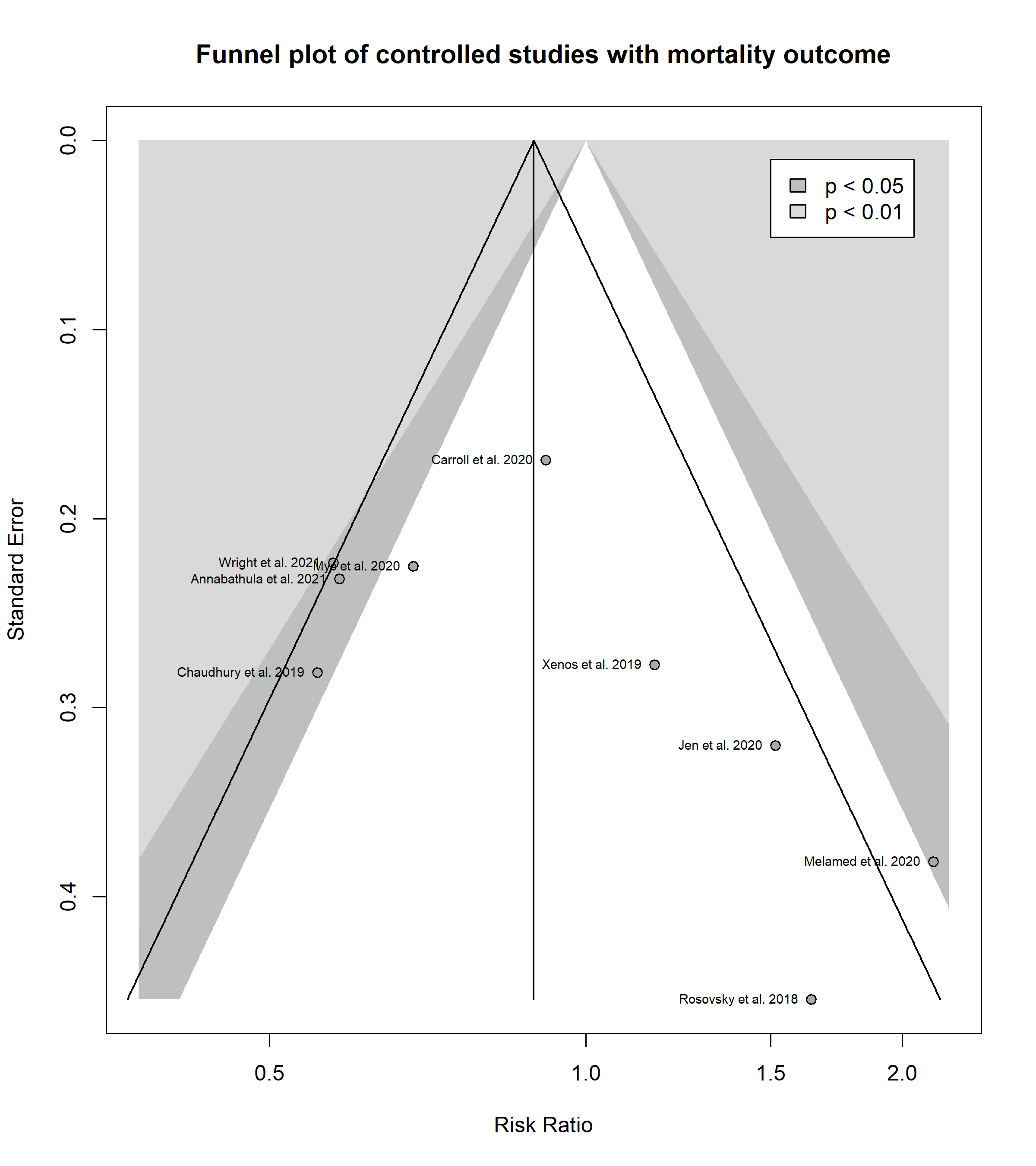
**
